# Comparison of early visual outcomes after SMILE using VISUMAX 800 and VISUMAX 500 for myopia: a retrospective matched case–control study

**DOI:** 10.1038/s41598-024-62354-y

**Published:** 2024-05-25

**Authors:** Tae Keun Yoo, Dongyoung Kim, Jung Soo Kim, Hee Sun Kim, Ik Hee Ryu, In Sik Lee, Jin Kuk Kim, Kun-Hoo Na

**Affiliations:** 1 Department of Ophthalmology, Hangil Eye Hospital, 35 Bupyeong-daero, Bupyeong-gu, Incheon, 21388 South Korea; 2 Department of Refractive Surgery, B&VIIT Eye Center, Seoul, South Korea; 3 Research and Development Department, VISUWORKS, Seoul, South Korea

**Keywords:** Corneal surgery, Laser, Refractive surgical procedure, Myopia, Vision, Outcomes research, Health care

## Abstract

VISUMAX 800 was introduced to improve the patient experience and clinical outcomes of small incision lenticule extraction (SMILE). This was a retrospective, matched, and case–control study (1:2) controlled for preoperative central corneal thickness and refractive error that compared early refractive and visual outcomes after SMILE using VISUMAX 800 and VISUMAX 500 to treat myopia. We included 50 eyes that underwent the VISUMAX 800 SMILE and 100 eyes that underwent the VISUMAX 500 SMILE. SMILE using VISUMAX 800 was performed using the CentraLign aid for vertex centration. Cyclotorsion was controlled by an OcuLign assistant in the VISUMAX 800 group after corneal marking. Corneal higher-order aberrations (HOAs) were evaluated using a Pentacam 1 month after surgery. No differences were observed in the pre- and post-operative refractive and visual outcomes at 1 day, 1 month, and 6 months after surgery. VISUMAX 800 induced less total HOAs than VISUMAX 500 (*P* = 0.036). No statistically significant differences were observed in the amounts of induced spherical aberrations or vertical and horizontal comas. No differences were observed in the 1 month and 6 months refractive and visual outcomes between two SMILE procedures, except for VISUMAX 800, which resulted in lower postoperative total HOAs than VISUMAX 500.

## Introduction

The worldwide prevalence of myopia and the use of laser vision correction has been increasing^1,2^. Small-incision lenticule extraction (SMILE) involves using a femtosecond laser to create an intrastromal lenticule with one incision in the cornea to correct refractive errors^3^. Since its introduction, SMILE performed using VISUMAX 500 (Carl Zeiss Meditec AG, Jena, Germany) has been an effective, predictable, and safe technique for correcting myopia. SMILE is a minimally invasive flapless procedure compared to laser in-situ keratomileusis (LASIK). SMILE has fewer dry eye symptoms and better postoperative corneal biomechanics than LASIK^4^.

However, SMILE using VISUMAX 500 has the disadvantage that the operator must manually match the patient's corneal vertex and docking center. Decentration using VISUMAX 500 can induce higher-order astigmatism, possibly related to reduced visual quality^5^. In addition, even if the cornea is marked to check the direction of astigmatism, it is difficult to correct cyclotorsion accurately after docking because the operator must manually rotate the eye contact device using VISUMAX 500. Decentration and a lack of cyclotorsion control can contribute to the under-correction of astigmatism^6^. Suction loss is another intraoperative complication with potential negative effects on postoperative outcomes^7^. Sudden eye movement during a VISUMAX 500 fs laser period of approximately 25–30 s causes suction loss. The incidence of suction loss in the SMILE procedure is approximately 0.4–2.7%^8,9^.

Recently, SMILE Pro® using VISUMAX 800 (Carl Zeiss Meditec AG, Jena, Germany) was introduced to improve patient experience and clinical outcomes. The upgraded femtosecond laser head of VISUMAX 800 offers a reduced refractive lenticule creation time of less than 10 s, which is significantly shorter than that of VISUMAX 500^10^. This shorter laser delineation time is expected to further reduce the possibility of suction loss. The system also provides additional modules, including a centration control aid (CentraLign) and cyclotorsion control aid (OcuLign), to assist operators^11^. These modules allow operators to accurately control surgical procedures performed manually based on their experience.

Only a few studies have reported the efficacy and safety of SMILE using VISUMAX 800^11,12^. In 2023, surgical results using VISUMAX 800 were reported for the first time^11^. In the same year, it was reported that SMILE could be completed faster using VISUMAX 800 than with VISUMAX 500^10^. In 2024, the results of using VISUMAX 800 were reported at a new vision correction center^12^. However, no study has compared the clinical outcomes of VISUMAX 800 and VISUMAX 500. A clinical comparison of VISUMAX 800, a new device that overcomes the shortcomings of VISUMAX 500, is required in a real-world setting. The present study compared early visual outcomes, including post-operative refractive errors and corneal higher-order aberrations (HOAs), after SMILE using VISUMAX 800 and VISUMAX 500.

## Materials and methods

### Study setting

This retrospective, matched case–control study (1:2) controlled for preoperative refractive errors. We retrospectively collected the preoperative measurements and post-operative outcome data from the B&V IIT Eye Center (Seoul, South Korea). To ensure independence of the measurement data, we randomly selected one eye from each patient for analysis. We included 50 eyes (50 patients) that underwent VISUMAX 800 SMILE and 100 eyes (100 patients) that underwent VISUMAX 500 SMILE for myopia and astigmatism correction between August and September 2023. As temperature and humidity can have an effect, data were collected for the same period in both groups. This study was approved by the Institutional Review Board of the Korean National Institute for Bioethics Policy (KNIBP; No. 2023-1219-001). The requirement for informed consent was waived due to the study’s retrospective nature. All research methods followed the Declaration of Helsinki and the KNIBP guidelines.

### Patient selection

The criteria for consideration in SMILE included the following: age ≥ 18 years; myopia with spherical equivalent ≥ − 10.0 diopters (D) and cylinder ≥ − 3.00 D; central corneal thickness (CCT) measured using pachymetry > 500 µm; residual corneal thickness > 380 µm after surgery; and absence of corneal abnormalities suggestive of keratoconus. All preoperative measurements were analyzed using an in-house screening system to identify candidate patients for safe surgery^13^. No differences were noted in the indications for using VISUMAX 800 and VISUMAX 500, and the surgical method was determined through sufficient consultation between the patient and surgeon. Because the institution where the study was conducted operated three VISUMAX 500 systems and one VISUMAX 800 system during the study period, the number of SMILE surgeries performed using the VISUMAX 500 system was overwhelmingly high. For each SMILE case using VISUMAX 800, two matched cases with the most similar manifest refractions were included in the VISUMAX 500 group. Therefore, the designed case–control ratio was 1:2 with the nearest-neighbor algorithm using the R software’s ‘MatchIt’ package in the standardized spaces of CCT, preoperative spheres, and cylinder^14^. Additionally, we manually confirmed that all case–control matching processes were performed based on the range of CCT measured using pachymetry within ± 20.0 µm and preoperative spheres and cylinders within ± 0.50 D in manifest refraction.

### Ocular measurements

All patients underwent full ophthalmic measurements during preoperative examinations, including uncorrected distance visual acuity (UDVA), corrected-distance visual acuity (CDVA), manifest refraction, slit-lamp examination, and dilated fundus examination^15^. Pachymetry (NT-530P; Nidek Co., Ltd., Aichi, Japan) was performed to evaluate central corneal thickness. A Pentacam Scheimpflug device (Oculus GmbH, Wetzlar, Germany) was used to measure corneal topography and HOAs 1 month after surgery. Corneal HOAs were evaluated under standard scotopic light settings in the 6.0 mm zone. The root mean square values of the total HOAs, individual Zernike coefficients of spherical aberration, and vertical and horizontal comas measured over the entire cornea were used in this study. Pentacam measurements were performed preoperatively and at the 1 month follow-up. Six months after surgery, only visual acuity and autorefraction were measured because of patient convenience issues. Experienced examiners performed all measurements.

### Surgical procedures

Three expert surgeons (HSK, IHR, and ISL) performed all the surgeries using the same techniques for each SMILE system. All experts were board-certified ophthalmologists with more than 10 years of experience in SMILE. We used the same customized nomogram developed based on a large electronic medical record database of SMILE surgery to target post-operative emmetropia for VISUMAX 800 and VISUMAX 500. For SMILE treatments using VISUMAX 800 and VISUMAX 500, we applied the same settings to the details of the refractive lenticule creation profiles. The diameter of the cap was 7.5 mm, and optical zone diameter was 6.2–6.7 mm. The intended cap thickness was 110–120 µm. A 2-mm incision was made at the 145° meridian.

In the VISUMAX 800 platform, the operators used the CentraLign aid function to display the corneal vertex and current treatment center. The position of the intraoperative corneal vertex relative to the center of the pupil was measured using preoperative Pentacam measurements. The operator controls the treatment center to the intraoperative corneal vertex. After centration using CentraLign, the cornea was fully attached to the docking device (disposable treatment package) using suction, and the OcuLign aid displayed the horizontal axis of the system. The operator used a joystick to align the preoperative corneal marks and OcuLign horizontal axis to control the cyclotorsion. The VISUMAX 500 platform has no assistant tools for centration or cyclotorsion control. The operator performed treatment centration based on the shape of the contact surface between the cornea and contact device using a VISUMAX 500. If there was a significant misalignment between the corneal marks and horizontal axis, the operator manually controlled the cyclotorsion with preoperative corneal marks by rotating the contact device (treatment package) on the VISUMAX 500 platform. If necessary, the eye and contact devices were docked again.

Surgery was performed using the same protocol for VISUMAX 800 and VISUMAX 500. Topical anesthesia was administered at the beginning of the surgery. The patients were instructed to focus on an internal light source during docking. After docking, centering and cyclotorsion controls were performed using VISUMAX 800 and VISUMAX 500. For lenticule extraction after laser delineation, the anterior interface of the lenticule was dissected, followed by the posterior interface. Once the lenticule was completely separated, it was removed from the cornea by using forceps. At the end of the surgery, the space under the cap was completely irrigated. After surgery, patients received a post-operative regimen that included moxifloxacin eye drops for 2 weeks and loteprednol eye drops for 4 weeks.

### Statistical analysis

We analyzed the differences in clinical measurements between the VISUMAX 800 and VISUMAX 500 groups. Comparisons between groups were performed using the chi-square test for categorical variables and Student’s t-test for quantitative variables. Post-operative astigmatism was evaluated using astigmatic vector analysis based on Alpin’s method^16^. In the VISUMAX 800 and VISUMAX 500 groups, the surgically induced astigmatism (SIA; actual change induced by surgery), target-induced astigmatism (TIA; intended change after surgery), and difference vector (the magnitude of astigmatism correction from the achieved result) were analyzed^17^. All statistical analyses were performed using the Statistical Package for the Social Sciences (SPSS; IBM Corp., Armonk, NY, USA), version 23.0. All statistical tests were two-sided with a significance level of *P* < 0.050.

The minimal sample size was calculated using a significance level of 5%, a power of 80%, and an enrollment ratio of 2.0. Prior data from the literature showed that the UDVA of the VISUAMX 800 was − 0.06 ± 0.08 logMAR. The UDVA of VISUMAX 500 was − 0.12 ± 0.11 logMAR at 1 month after surgery. The calculated sample size to identify differences in visual outcomes was 120 (40:80). Therefore, the collected data are statistically sufficient to provide reliable conclusions based on this calculation.

## Results

### Demographics and preoperative data

In this study, we analyzed 50 eyes of 50 patients who underwent SMILE using VISUMAX 800 and 100 eyes of 100 patients who underwent SMILE using VISUMAX 500. All the surgeries were performed without intraoperative complications. We observed that all cases using VISUMAX 800 had a laser duration of less than 10–11 s, whereas the laser duration of VISUMAX 500 was 27–30 s (Supplementary Fig. 1). Three surgeons (HSK, IHR, and ISL) performed 20, 18, and 12 surgeries using VISUAMX 800 and 58, 26, and 16 surgeries using VISUMAX 500. No significant differences were observed in postoperative outcomes between the surgeons (data not shown). No post-operative complications occurred during the follow-up period in both groups. Demographic and preoperative measurements are shown in Table 1. No significant differences were observed in age, sex, CCT findings, manifest refraction, or CDVA between the VISUMAX 800 and 500 groups.

**Table 1:** Demographics and preoperative measurements for SMILEs using VISUMAX 800 and VISUMAX 500.

	VISUMAX 800	VISUMAX 500	*P* value
Number of eyes (Number of patients)	50 (50)	100 (100)	N/A
Age (year)	24.5 ± 5.3 [range, 19 to 39]	25.1 ± 5.4 [range, 19 to 38]	0.663
Gender (%, female)	30 (60%)	56 (56%)	0.743
Central corneal thickness (μm)	557.2 ± 21.4 [range, 512 to 590]	555.1 ± 24.6 [range, 509 to 609]	0.615
Preoperative MR sphere (D)	− 3.63 ± 1.42 [range, − 6.75 to − 1.12]	− 3.61 ± 1.15 [range, − 6.87 to − 1.25]	0.911
Preoperative MR cylinder (D)	− 0.78 ± 0.53 [range, − 2.25 to − 0.25]	− 0.78 ± 0.63 [range, − 2.25 to 0.00]	0.998
Preoperative CDVA (logMAR)	0.006 ± 0.024 [range, 0.0 to 0.1]	0.004 ± 0.020 [range, 0.0 to 0.1]	0.587

CDVA, corrected distance visual acuity; logMAR, logarithm of the minimum angle of resolution; MR, manifest refraction.

### Summary of clinical outcomes

**Figure 1:**
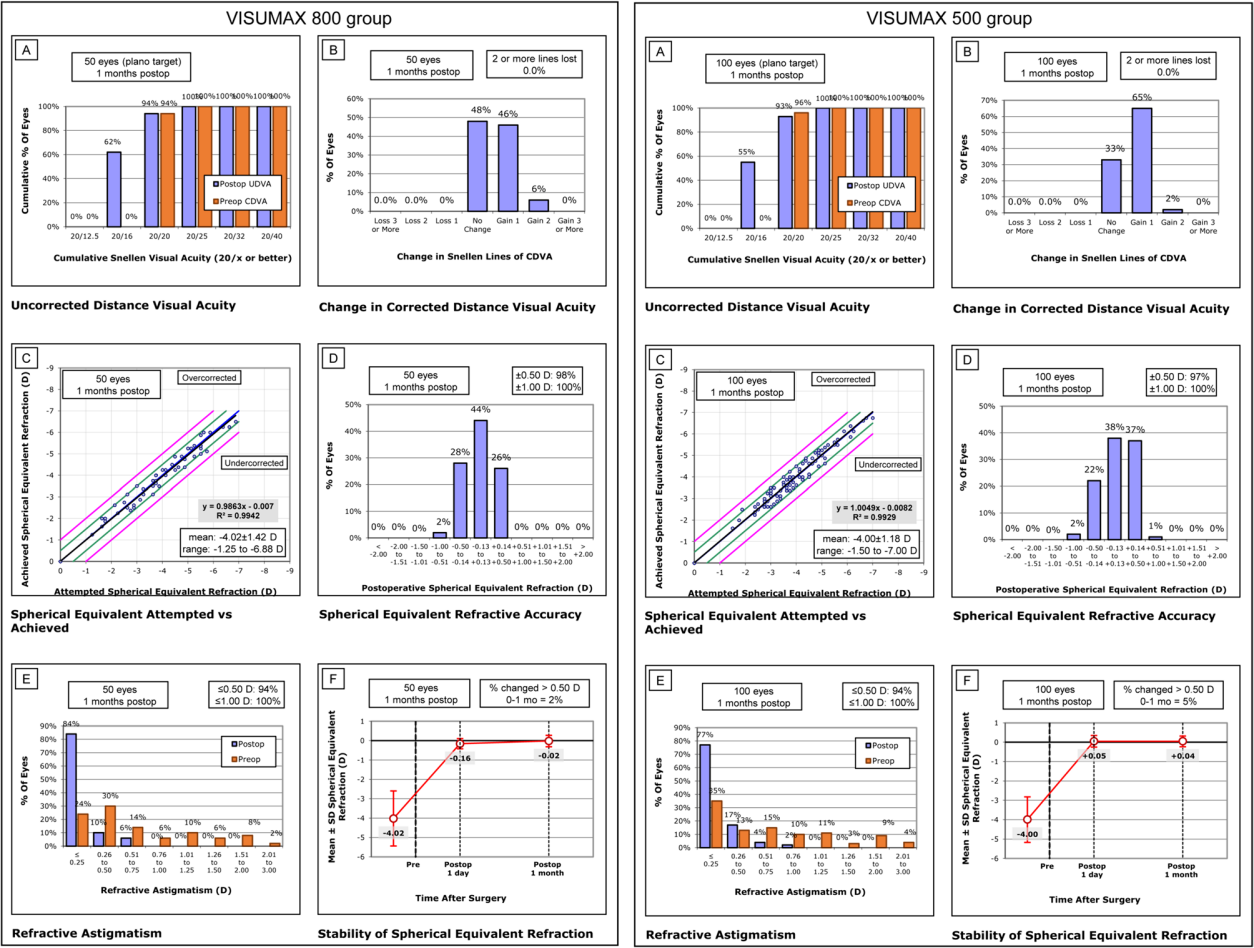
Clinical outcomes of SMILEs using VISUMAX 800 and VISUMAX 500 at 1 month follow-up. (**A**) Uncorrected-distance visual acuity (UDVA). (**B**) Change in corrected-distance visual acuity (CDVA). (**C**) Attempted vs achieved spherical equivalent (manifest refraction). (**D**) Spherical-equivalent refractive accuracy (manifest refraction). (**E**) Refractive astigmatism (manifest refraction). (**F**) Stability of spherical-equivalent refraction (automated refraction).

Figure 1 shows the clinical outcomes of the VISUMAX 800 and VISUMAX 500 groups at 1 month follow-up. The distribution of post-operative UDVA showed no significant differences between the groups (*P* = 0.716 using the chi-square test). The distributions of the post-operative spherical equivalent and cylinder were not significantly different between the groups (*P* = 0.643 for the spherical equivalent and *P* = 0.457 using the chi-square test). All eyes showed the same or better post-operative UDVA than preoperative CDVA in both groups. After surgery, a change in refractive errors of 0.5 D or more was observed in one eye in the VISUMAX 800 group and five eyes in the VISUMAX 500 group (*P* = 0.378).

### Comparison results of visual acuity and refractions

**Figure 2:**
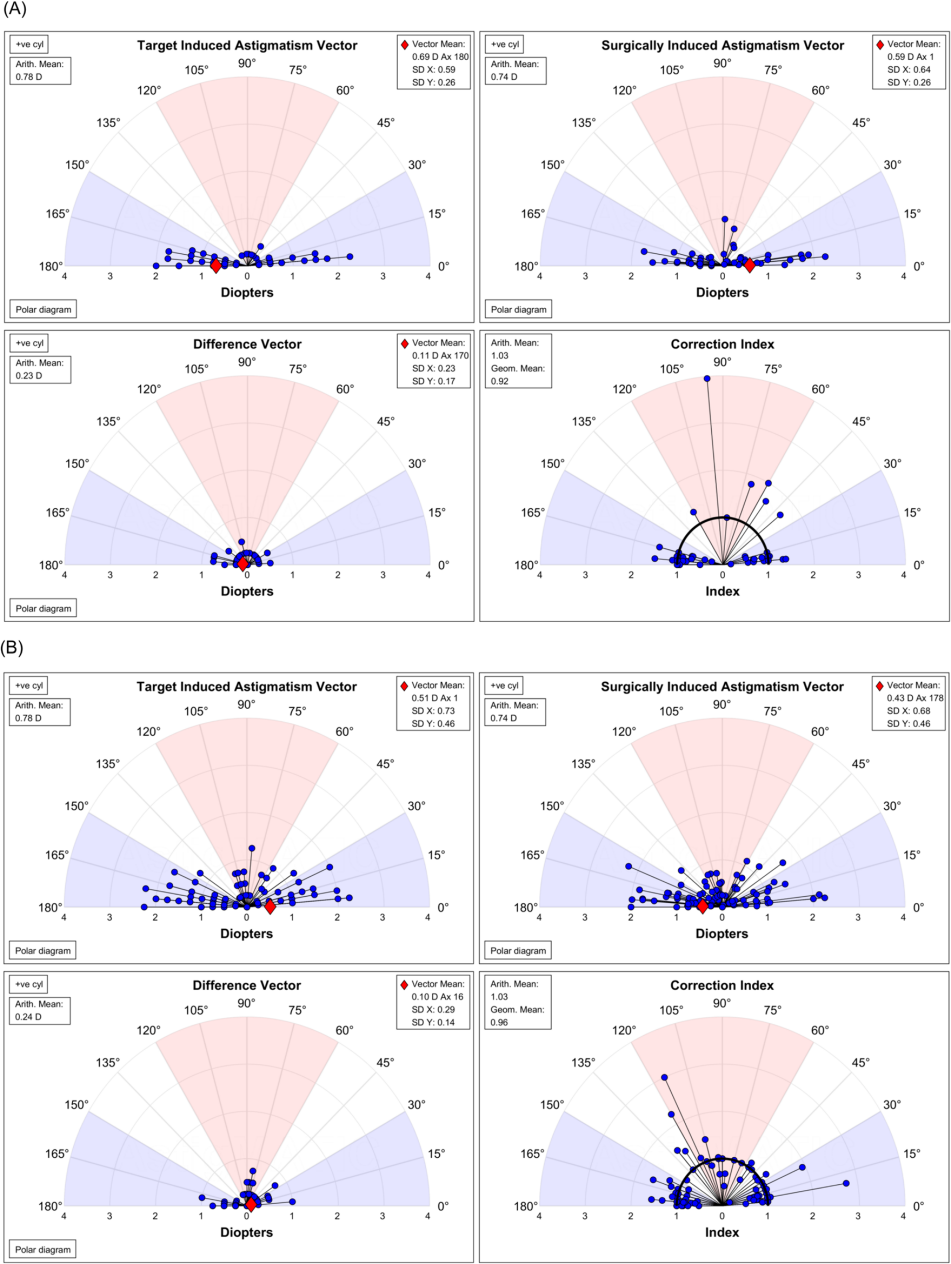
Alpins vector analysis of the effectiveness of astigmatism correction using VISUMAX 800 and VISUMAX 500. (**A**) VISUMAX 800 group. (**B**) VISUMAX 500 group.

Table 2 compares the post-operative visual and refractive outcomes. No significant differences were observed in the post-operative 1 day (*P* = 0.738) and 1 month (*P* = 0.591) logMAR UDVA values. No significant difference was observed in post-operative 6 month UDVA (*P* = 0.741). Although 1 day sphere values for automated refraction (*P* = 0.007) and manifest refraction (*P* = 0.004) showed significant differences, 1 month sphere values showed no significant differences for automated refraction (*P* = 0.355) and manifest refraction (*P* = 0.154). The cylinder values for automated and manifest refractions showed no differences across the follow-up periods. Six months after surgery, the sphere and cylinder values in automated refraction showed no significant differences (*P* = 0.138 for spheres and *P* = 0.441 for cylinders). The vector analysis results using post-operative 1 month manifest refraction are shown in Fig. 2. In the Alpins vector analysis, no significant differences were observed in SIA (*P* = 0.948), TIA (*P* = 0.932), difference vector (*P* = 0.890), or correction index (*P* = 0.915) between the VISUMAX 800 and VISUMAX 500 groups.

**Table 2:** Post-operative visual acuity and refractive measurements for SMILEs using VISUMAX 800 and VISUMAX 500 at 1 day, 1 month, and 6 months after surgery.

	VISUMAX 800	VISUMAX 500	*P* value
Visual acuity			
Day 1 UDVA (logMAR)	− 0.024 ± 0.077 [range, − 0.10 to 0.20]	− 0.019 ± 0.091 [range, − 0.10 to 0.30]	0.738
Month 1 UDVA (logMAR)	− 0.056 ± 0.061 [range, − 0.10 to 0.10]	− 0.050 ± 0.066 [range, − 0.10 to 0.20]	0.591
Month 6 UDVA (logMAR)	− 0.020 ± 0.042 [range, − 0.10 to 0.10]	− 0.027 ± 0.058 [range, − 0.10 to 0.10]	0.741
ARK data			
Day 1 AR sphere (D)	− 0.42 ± 0.41 [range, − 2.00 to + 0.25]	− 0.24 ± 0.39 [range, − 1.50 to + 0.50]	0.007
Day 1 AR cylinder (D)	− 0.38 ± 0.27 [range, − 1.00 to 0.00]	− 0.39 ± 0.23 [range, − 1.00 to 0.00]	0.725
Month 1 AR sphere (D)	− 0.20 ± 0.54 [range, − 1.75 to + 1.00]	− 0.13 ± 0.40 [range, − 1.25 to + 0.75]	0.355
Month 1 AR cylinder (D)	− 0.39 ± 0.23 [range, − 1.00 to 0.00]	− 0.40 ± 0.25 [range, − 1.25 to 0.00]	0.861
Month 6 AR sphere (D)	− 0.23 ± 0.59 [range, − 0.75 to + 0.75]	− 0.11 ± 0.35 [range, − 0.75 to + 0.75]	0.138
Month 6 AR cylinder (D)	− 0.33 ± 0.24 [range, − 0.75 to 0.00]	− 0.38 ± 0.20 [range, − 0.75 to 0.00]	0.441
MR data*			
Day 1 MR sphere (D)	− 0.04 ± 0.28 [range, − 0.75 to + 0.50]	+ 0.13 ± 0.31 [range, − 0.37 to + 1.25]	0.004
Day 1 MR cylinder (D)	− 0.24 ± 0.24 [range, − 0.75 to 0.00]	− 0.17 ± 0.19 [range, − 0.75 to 0.00]	0.076
Month 1 MR sphere (D)	+ 0.09 ± 0.29 [range, − 0.50 to + 0.75]	+ 0.16 ± 0.30 [range, − 0.25 to + 0.75]	0.154
Month 1 MR cylinder (D)	− 0.23 ± 0.20 [range, − 0.75 to 0.00]	− 0.24 ± 0.23 [range, − 1.25 to 0.00]	0.650

ARK, automated refraction; logMAR, logarithm of the minimum angle of resolution; MR, manifest refraction; UDVA, uncorrected distance visual acuity.

*For patient convenience, MR examinations were not performed at 6 months after surgery.

### Comparison results of HOAs

**Figure 3:**
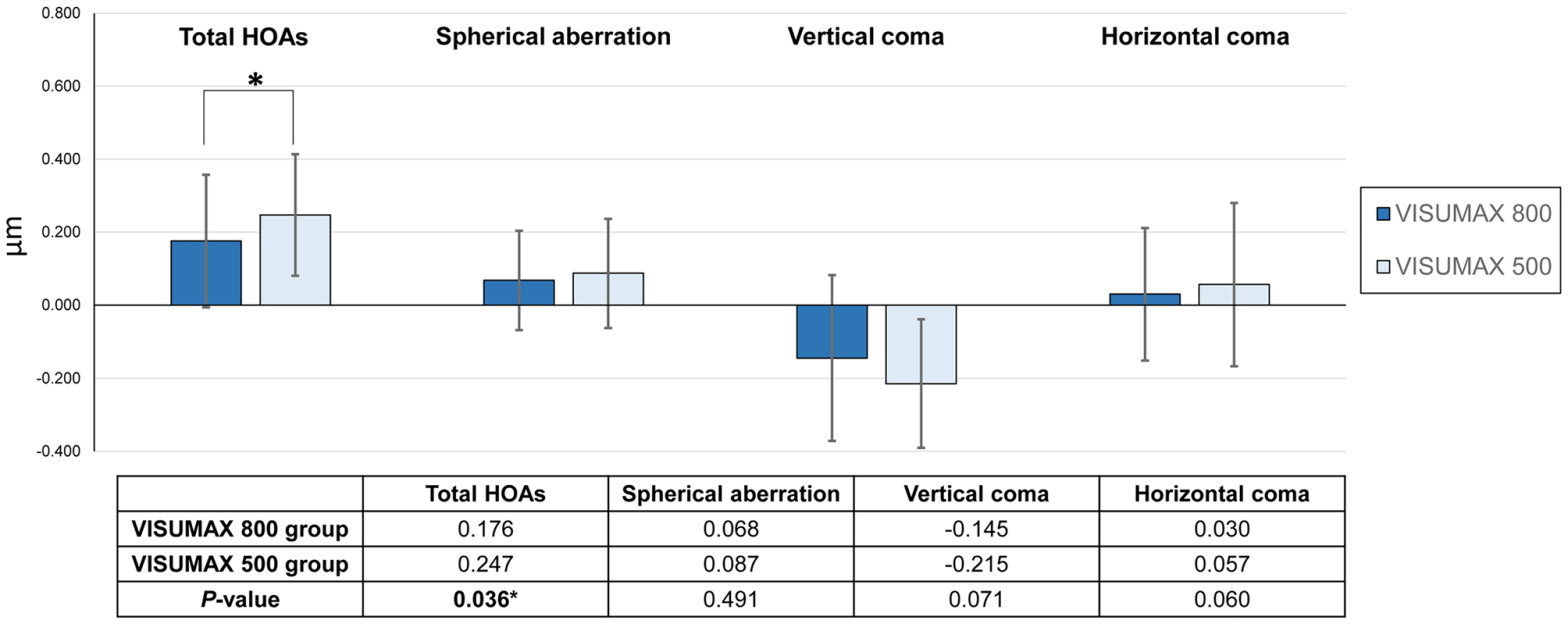
Amount of induced higher-order aberrations (HOAs) of SMILEs using VISUMAX 800 and VISUMAX 500. The total amount of induced HOAs was determined by subtracting the preoperative HOAs values from the 1 month postoperative HOAs values.

Table 3 compares the preoperative and post-operative HOAs between groups. None of the groups’ preoperative HOAs values differed significantly (*P* > 0.05). One month postoperatively, the VISUMAX 800 group (total HOAs = 0.461 μm) showed a significantly lower total HOAs (*P* = 0.037) than the VISUMAX 500 group (total HOAs = 0.525 μm). Figure 3 shows that the induction of total HOAs was significantly lower (*P* = 0.036) in the VISUMAX 800 group (induction of total HOAs = 0.176 μm) than that in the VISUMAX 500 group (induction of total HOAs = 0.247 μm). The induction of individual Zernike coefficients, including spherical aberration and horizontal and vertical coma, showed lower values in the VISUMAX 800 group than those in the VISUMAX 500 group, but with no statistical significance (*P* = 0.491 for spherical aberration, *P* = 0.071 for vertical coma, and *P* = 0.060 for horizontal coma).

**Table 3:** Preoperative and post-operative 1 month higher-order aberrations (HOAs) for SMILEs using VISUMAX 800 and VISUMAX 500.

	VISUMAX 800	VISUMAX 500	*P* value
Preoperative HOAs			
Total HOAs (μm)	0.285 ± 0.093	0.278 ± 0.105	0.730
Spherical aberration (μm)	0.188 ± 0.099	0.158 ± 0.076	0.079
Vertical coma (μm)	0.024 ± 0.166	− 0.025 ± 0.204	0.182
Horizontal coma (μm)	0.001 ± 0.102	− 0.001 ± 0.137	0.948
Month 1 post-operative HOAs			
Total HOAs (μm)	0.461 ± 0.151	0.525 ± 0.160	0.037
Spherical aberration (μm)	0.256 ± 0.140	0.245 ± 0.154	0.717
Vertical coma (μm)	− 0.121 ± 0.259	− 0.240 ± 0.263	0.046
Horizontal coma (μm)	0.031 ± 0.176	0.056 ± 0.264	0.052

HOAs, higher-order aberrations.

## Discussion

### Contribution of this study

It is questionable whether the advantages of VISUMAX 800 are sufficient for clinical performance^18^. Therefore, this study compared the clinical results of VISUMAX 800 and VISUMAX 500 SMILE. No significant difference was observed in visual acuity and refractive power, and VISUMAX 800 showed better results regarding the induction of total HOAs. This might be due to the more accurate centration using CentraLign of VISUMAX 800. However, each detailed item, including the amounts of induced spherical aberration and horizontal and vertical comas, showed no significant difference.

SMILE has similar or superior vision improvement effects and predictive power to LASIK^19,20^. VISUMAX 800 began to be used in Europe in the second half of 2021 after the Conformité Européenne (CE) certification. In Korea, VISUMAX 800 was commercially used from the second half of 2023. Although a previous study reported safe and effective clinical outcomes for correcting myopia^11^, no study has compared the clinical results of SMILE using VISUMAX 800 and VISUMAX 500. To our knowledge, this is the first study to compare clinical visual outcomes and corneal HOAs after SMILE using VISUMAX 800 and VISUMAX 500. Because many clinics are curious about the performance of the new femtosecond laser system for SMILE (i.e., VISUMAX 800), we report its early clinical results to confirm its safety and efficacy compared to the previous version of the laser system, VISUMAX 500. Vision correction using both devices showed good clinical results and was predictable and safe.

### Advantages of VISUMAX 800 compared to VISUMAX 500

In the last decade, the introduction of SMILE has provided a safe, effective, and predictable option for patients undergoing refractive surgery with rapid post-operative recovery^21^. SMILE causes lower dry eye and HOAs, and has better biomechanical properties than LASIK^22^. However, in the docking, centration, and axis adjustment processes using VISUMAX 500, many depend on the operator’s experience^23^. Owing to the relatively long laser duration of VISUMAX 500, uncooperative and anxious patients should be excluded as candidates for SMILE to avoid complications of potential suction off^24^. A new femtosecond laser system, VISUMAX 800, provides a faster laser for lenticule creation and assistive tools for centration and cyclotorsion control^10^. The significant advantage that operators can experience with VISUMAX 800 is its fast laser time. In this study, we confirmed that a laser time of approximately 30 s was required when VISUMAX 500 was used. In contrast, laser lenticule formation was completed within 11 s when VISUMAX 800 was used.

Our study shows that the VISUMAX 800 has the added advantage of lower total HOAs. The less HOAs induction was due to the user interface of the VISUMAX 800, which quantitatively shows the position of the astigmatism axis and vertex center intraoperatively. Analyzing the topographic shape of the cornea and adjusting the preoperative astigmatism are very important in SMILE to obtain good clinical results^25^. However, these processes can be disrupted by an asymmetric corneal surface or dry eye conditions^26^. Centration and axis parameters, which previously depended on the operator’s manual adjustment and experience with the VISUMAX 500, can now be quantitatively adjusted based on ocular measurements such as corneal topography in the VISUMAX 800.

### Comparison of clinical outcomes between VISUMAX 800 and VISUMAX 500

In our study, the VISUMAX 800 and VISUMAX 500 groups showed no significantly different outcomes in post-operative 1 month visual acuity, refractive errors, or astigmatism vector analysis. Additionally, post-operative 6 months visual acuity and refractive errors showed no significant differences. This study was conducted by experienced operators who successfully controlled the cyclotorsion using manual procedures and postural alignment on the VISUMAX 500 platform. Therefore, the effect of the additional astigmatism correction of the new OcuLign system in VISUMAX 800 was nonsignificant. Cross-axis alignment in SMILE may significantly reduce the under-correction of astigmatism^27^. The OcuLign aid may be helpful in severe myopic astigmatism correction or for novice operators.

One day after surgery, the VISUMAX 800 group showed a greater myopic tendency than the VISUMAX 500 group; however, this resolved after 1 month. This may be because using OcuLign on VISUMAX 800 requires more marks on the corneal epithelium to draw the astigmatism axis. Additionally, this may be because the surgeons were not familiar with VISUMAX 800, which resulted in more unnecessary manipulations than expected. VISUMAX 800 was designed to have optics and laser delivery systems identical to those of VISUMAX 500^11^. Surgery using the two femtosecond laser systems was performed using the same optical principle and surgical sequence. Therefore, it was expected that the two clinical outcomes would not differ between experienced operators. As the predictability of the visual outcomes of SMILE using VISUMAX 500 has been well-established by short- and long-term studies^28^, VISUMAX 800 was expected to show good predictability during long-term follow-up.

### Comparison of HOAs between VISUMAX 800 and VISUMAX 500

Our findings showed that VISUMAX 800 induced less HOAs in SMILE than VISUMAX 500. However, no difference was observed in the change in the individual Zernike coefficients of the spherical aberration and vertical and horizontal comas. No significant difference was observed in the individual factors; however, when all of them accumulated, a significant improvement was observed in the VISUMAX 800 group. This may be due to the new functions of the VISUMAX 800, CentraLign, and OcuLign. Generally, higher HOAs are associated with lower visual quality after refractive surgery^29^. Decentration was related to a larger induction of HOAs in SMILE^30^. In a previous study comparing binocular SMILE data, more centered eyes showed better clinical outcomes in terms of HOAs^31^. Our results showed that CentraLign of VISUMAX 800 significantly helped the operators align the center of the optical zone and improved the clinical outcomes in HOAs compared to VISUMAX 500 (Supplementary Fig. 2). Because there is no standard technique to align the center of the optical zone in SMILE using VISUMAX 500, there is a risk of decentered lenticule formation, which can lead to unfavorable outcomes^6^. Operators must rely on tear film marks for centration using VISUMAX 500, which are difficult to trust because of factors such as dryness and corneal shape^32^. The irregular or asymmetric shape of the corneal surface in VISUMAX 500 can cause decentration, leading to a larger induction of HOAs^26^. Occasionally, unsatisfactory postoperative visual acuity results in SMILE may result from this issue^33^. CentraLign, a new assistant function of VISUMAX 800, provided accurate and reliable centration with intraoperative visual assistance and demonstrated the ability to block the possibility of decentration.

### Strengths and limitations of this study

The strengths of this study include the matched case–control comparison between the VISUMAX 800 and VISUMAX 500 groups. However, this study had some limitations. First, the eye selection for surgery was not performed using a randomized controlled design. Therefore, possible variability should be considered when interpreting the results of our study, although we attempted to match preoperative CCT and refractive errors. Secondly, the small number of SMILE cases may have raised concerns regarding the statistical power of the comparative analysis. Although this study was conducted using a case–control comparison, it is currently unclear whether the number of patients was sufficient to confirm the differences in clinical outcomes. Third, this study was conducted at a single clinic in East Asia, which raises uncertainty regarding the generalizability of our findings to other clinics and ethnic groups. To confirm our findings, a multicenter study with a larger sample size is warranted. Finally, the short follow-up period is a major limitation. However, 1 month is sufficient for the HOAs analysis because rapid recovery has been reported after SMILE^21,22^. To overcome this, we additionally tracked and reported the patients’ visual acuity and automated refraction values at 6 months after surgery. In addition, because studies on clinical outcomes using VISUMAX 800 are currently too scarce, the findings of the present study are clinically and academically important.

## Conclusion

No discernible difference was observed in the early visual outcomes of visual acuity and refractive error between the SMILE surgeries using VISUMAX 800 and VISUMAX 500. A comparative analysis showed that VISUMAX 800 resulted in a lower induction of total HOAs than VISUMAX 500. This difference may be due to the more accurate centration using CentraLign in VISUMAX 800. No significant differences in the were observed 1 month and 6 months other refractive outcomes between the two groups. Both VISUMAX 800 and VISUMAX 500 showed high efficacy and safety in myopia correction. The new assistant tools and fast laser duration in VISUMAX 800 may be helpful, especially for novice operators who have difficulty centering and correcting cyclotorsion. Further multicenter studies with long-term follow-ups are required to confirm our findings.

### Supplementary Information

Supplementary Figures.

## Data Availability

Tae Keun Yoo had full access to all the data in the study and takes responsibility for the integrity of the data and accuracy of the data analysis. The data used in this study cannot be made publicly accessible due to restrictions from the Ethics Committee of the Institutional Review Board of the Korean National Institute for Bioethics Policy (KNIBP; No. 2023–1219-001). Data can be accessed upon reasonable request pending approvals from the Ethics Committees. The dataset used in this study may be obtained from the corresponding author upon reasonable request.
