# Supplementary material for: Comparison of early visual outcomes after SMILE using VISUMAX 800 and VISUMAX 500 for myopia: a retrospective matched case–control study
Source: Sci Rep. 2024 May 25;14:11989. doi: 10.1038/s41598-024-62354-y (PMC11127987; doi:10.1038/s41598-024-62354-y)
Supplement: Supplementary file 1 — Supplementary Figures. [file 41598_2024_62354_MOESM1_ESM.pdf]

Comparison of early visual outcomes after SMILE using VISUMAX 800 and VISUMAX 500 for myopia: a retrospective matched case-control study

Supplementary materials

Supplementary Figure 1. Treatment reports of SMILE to find the laser duration. (A) 11 s in VISUMAX 800. (B) 30 s in VISUMAX 500.

Supplementary Figure 2. Comparison of two cases with VISUMAX 800 and VISUMAX 500.

(A) VISUMAX 800 (B) VISUMAX 500

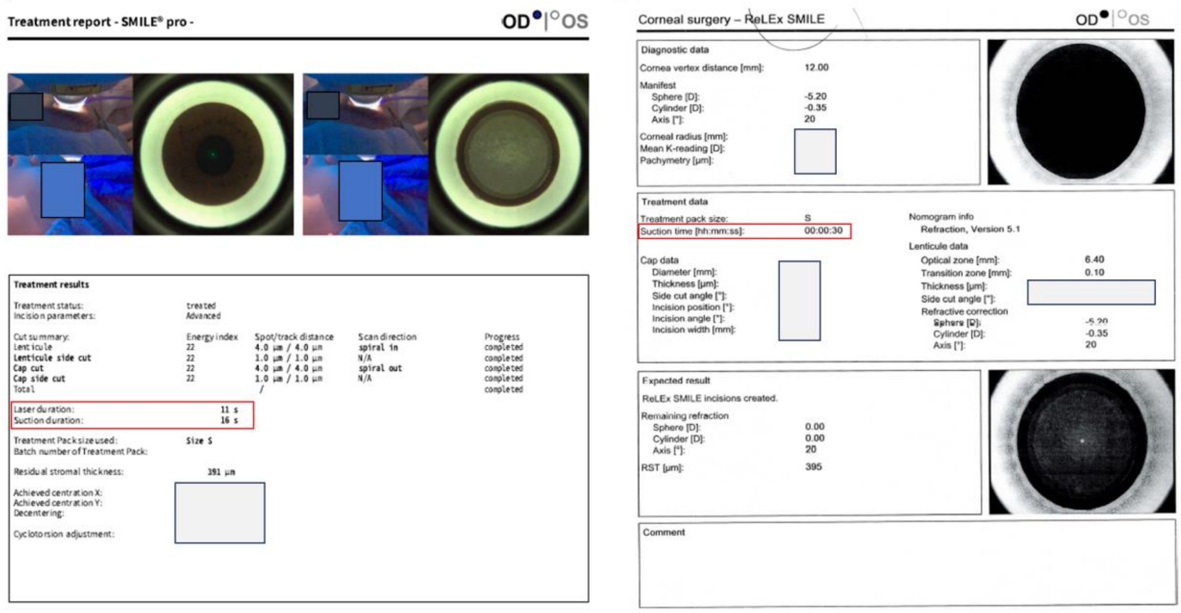

Supplementary Figure 1. Treatment reports of SMILE to find the laser duration. (A) 11 s in VISUMAX 800. (B) 30 s in VISUMAX 500.

Case A – VISUMAX 800

Preop MR
	SPH	CYL	AX
R:	-1.50	-0.50	5
L:	-1.50	-0.50	0

Postop 1M MR
	SPH	CYL	AX	VA
R:	+0.25	0.00	0	1.2
L:	+0.25	-0.25	5	1.2

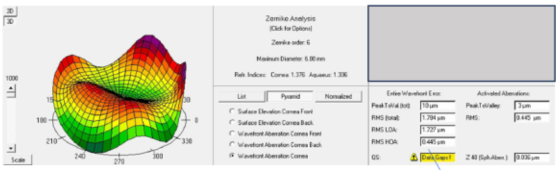

Case B – VISUMAX 500

Preop MR
	SPH	CYL	AX
R:	-1.50	-0.50	165
L:	-1.50	-0.25	5

Postop 1M MR
	SPH	CYL	AX	VA
R:	+0.25	0.00	0	1.2
L:	+0.25	0.00	0	1.2

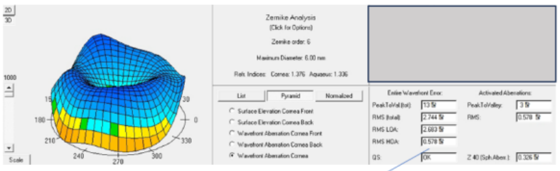

		PreopHOA_RMS				PostopHOA_RMS				**Postop-Preop difference**			
		Total HOA	Spherical Aberration	Coma Vertical	Coma Horizontal	Total HOA	Spherical Aberration	Coma Vertical	Coma Horizontal	**Total HOA**	Spherical Aberration	Coma Vertical	Coma Horizontal
**Case A**	OD	0.359	0.135	-0.161	-0.177	0.445	0.036	-0.321	-0.178	**0.086**	-0.099	-0.16	-0.001
**VISUMAX 800**	OS	0.364	0.157	-0.197	0.164	0.468	0.168	-0.141	0.261	**0.104**	0.011	0.056	0.097
**Case B**	OD	0.27	0.153	-0.05	0.003	0.578	0.326	-0.427	-0.043	**0.308**	0.173	-0.377	-0.046
**VISUMAX 500**	OS	0.589	0.23	-0.451	-0.015	0.818	0.303	-0.485	0.164	**0.229**	0.073	-0.034	0.179

Supplementary Figure 2. Comparison of two cases with VISUMAX 800 and VISUMAX 500.
